# Sequencing complete mitochondrial genome of *Lanius sphenocercus sphenocercus* (Passeriformes: Laniidae) using Illunima HiSeq 2500

**DOI:** 10.1080/23802359.2016.1167640

**Published:** 2016-04-19

**Authors:** Mei-Xia Yang, Qing-Xiong Wang, Hong Xiao, Chao Yang

**Affiliations:** a Shaanxi Institute of Zoology, Xi’an, China;; b School of Life Sciences, Shaanxi Normal University, Xi’an, China

**Keywords:** High-throughput sequencing, *Lanius sphenocercus*, mitogenome, phylogeny

## Abstract

Using an Illumina platform, we sequenced the complete mitochondrial genome of *Lanius sphenocercus sphenocercus* to an average coverage of 1669.5×. We performed a *de novo* assembly using SOAPdenovo2 and obtained the total mitogenome with 16,833 bp in length. Most PCGs begin with the typical ATG start codon with the exception of *COI* gene, which use GTG as the initiation codon. Stop codons AGG, TAG, TAA, and AGA are present in the PCGs; exceptions are *COII, COIII* and *ND4*, which possess incomplete termination codons (T). But, the function of *COII* with incomplete stop codon T should be further investigated. The phylogeny revealed that genetic distance of Laniidae and Corvidae was closer than other species. Compared to other three shrike species, *L. s. sphenocercus* occupy a separate status in the genus *Lanius.*

Chinese Grey Shrike (*Lanius sphenocercus*) is an endemic species in Eastern Asia. It belongs to the compact group of gray shrikes and with two subspecies: nominate subspecies (*Lanius sphenocercus sphenocercus*) and south-west subspecies (*Lanius sphenocercus giganteus*) in China. At present, most studied species are the groups that inhabit Europe and North America. Data on the biology, vocalization, and behaviour of Asian species of the group are scarce (Opaev 2014). Especially, the limited molecular data dampen the evolution and diversity studies in genus *Lanius.*

A naturally dead *L. s. sphenocercus* was collected at Hongjian Nur (39°04'N, 109°53'E), Shaanxi, China. The specimen (Voucher number: XWBL01) was preserved in 100% ethanol and stored at −20 °C, and deposited in the animal specimens museum of Shaanxi Institute of Zoology, Xi’an, China. Genomic DNA was prepared in paired-end libraries, tagged and subjected to the high-throughput Illumina HiSeq2500 platform with 125bp paired-end strategy and yield 23,398,290 Paired-End Raw Reads with 126 bp in length. Clean reads were trimmed by removing regions with a Phred score of <10. Mapping against the complete mitogenome of *Lanius isabellinus* (GenBank: KP995437), high-quality reads were assembled using SOAPdenovo2 (Hahn et al. 2013). A total of 228,309 individual mitochondrial reads gave an average coverage of 1669.5×. Comparing with the *L. isabellinus*, annotations were generated in MITOchondrial genome annotation Server (MITOS) (Bernt et al. 2013).

The total length of *L. s. sphenocercus* mitogenome is 16,833 bp (GenBank: KU884610). The base composition of the entire mitogenome was 30.5% A, 25.4% T, 29.0% C and 15.1% G. There are 17 intergenic spacer regions, ranging in size from 1 to 10 bp (81 bp in total). Gene overlap involves 29 bp over 6 locations.

The typical ATG start codon is present in 12 of the 13 *L. s. sphenocercus* PCGs, nonstandard start codons (GTG) are found in *COI*. Open-reading frames of most *L. s. sphenocercus* genes end with AGG, TAA, AGA, or TAG, while *COII*, *COIII* and *ND4* have the incomplete stop codon T. Particularly, the *COII* with incomplete T merely exists in Laniidae of Aves and the function is still needed to be confirmed.

The two rRNA genes, 973 bp in *srRNA* and 1603 bp in *lrRNA*, are located between *tRNA^Phe^* and *tRNA^Leu^(UUR)* and separated by *tRNA^Val^* gene. All tRNA genes, ranging from 66 to 75 bp in size, can fold into a typical cloverleaf secondary structures, with exception of the *tRNA^Ser^(AGY)* (66 bp) which lacks the DHU arm. The control region was located between *tRNA^Glu^* and *tRNA^Phe^* with 1263 bp in length and 58.5% A + T content.

To investigate the phylogenetic position of *L. s. sphenocercus*, a Bayesian tree was reconstructed based on 13 mitogenomes using MrBayes ver. 3.2.2 (Ronquist et al. 2012) under the GTR + I + G model. *Pterodroma brevirostris* (GenBank: AY158678) was selected as an outgroup (Liang et al. 2007). The phylogram obtained from Bayesian inference (BI) strongly supported that Laniidae has the closest relationship with Corvidae. In the genus *Lanius*, *L. s. sphenocercus* is classified as Grey Shrikes which with high genetic similarities and morphological characteristics and clearly differentiated from other shrike species (Klassert et al. 2008; Olsson et al. 2010) (Figure 1).

**Figure 1.**
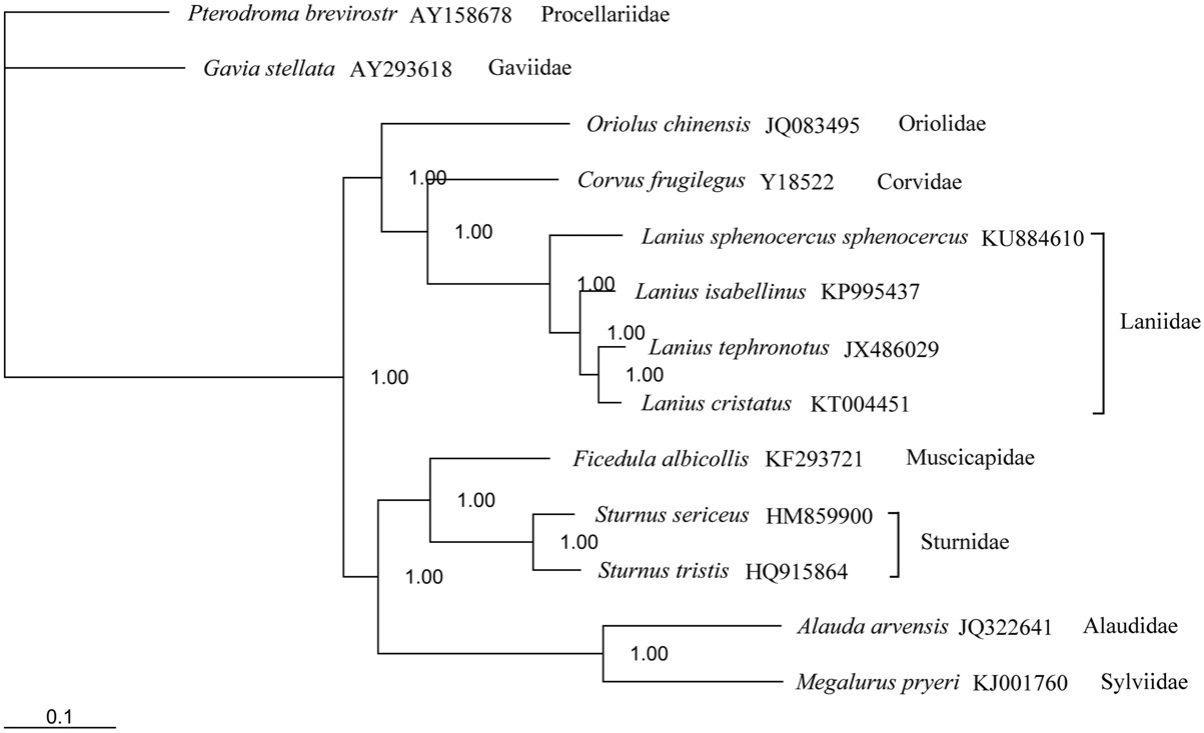
Topology of Bayesian tree for 13 species based on mitogenome sequences. Above the branches are Bayesian posterior probabilities values. GenBank accession numbers are indicated following species name.
